# Long non-coding RNA LINC00958 promotes colorectal cancer progression by enhancing the expression of LEM domain containing 1 via microRNA miR-3064-5p

**DOI:** 10.1080/21655979.2021.1985259

**Published:** 2021-10-21

**Authors:** Zhaoxia Luo, Shunxin Hao, Jian Yuan, Kai Zhu, Shuo Liu, Jing Zhang, Lei Yao

**Affiliations:** Department of General Surgery, Tianyou Hospital Affiliated to Wuhan University of Science and Technology, Wuhan, China

**Keywords:** LINC00958, miR-3064-5p, LEMD1, colorectal cancer, apoptosis, migration, invasion

## Abstract

Colorectal cancer is a common cause of cancer-related death worldwide. Thus, there is an urgent need to determine the mechanism of progression of colorectal cancer. In this study, we investigated the function and mechanism of long non-coding RNA LINC00958, providing a new biomarker for colorectal cancer. The expression of LINC00958, miR-3064-5p, and LEM domain containing 1 (LEMD1) in colorectal cancer tissues and cell lines was analyzed using reverse transcription quantitative polymerase chain reaction (RT-qPCR). The interaction between LINC00958, miR-3064-5p, and LEMD1 was assessed using the luciferase assay. The viability, proliferation, migration, invasion, and apoptosis of colorectal cancer cells with silenced LINC00958, miR-3064-5p, and LEMD1 were investigated using the cell counting kit-8 (CCK-8), 5′-Bromo-2′-deoxyuridine (BrdU), flow cytometry, wound healing, and transwell assays. Phosphorylated phosphoinositide 3-kinase (p-PI3K) and phosphorylated protein kinase B (p-AKT) protein levels were measured by western blotting. LINC00958 and LEMD1 were found to have increased, while the expression of miR-3064-5p was decreased in colorectal cancer tissues and cell lines. Silencing of LINC00958 hampered cell viability, proliferation, migration, and invasion, while enhancing the apoptosis in colorectal cancer cells. Notably, LINC00958 inhibited miR-3064-5p and promoted LEMD1; the miR-3064-5p inhibitor abrogated the effect of LINC00958 silencing in colorectal cancer cells. Additionally, LEMD1 knockdown inhibited the activation of PI3K/AKT signaling. Our analyses have shown that LINC00958 could facilitate the progression of colorectal cancer by sponging miR-3064-5p and releasing LEMD1, leading to the activation of the PI3K/AKT pathway. Thus, LINC00958 may be considered as an effective biomarker for the treatment of colorectal cancer.

## Introduction

Colorectal cancer is a digestive system malignancy with a high prevalence and death rate worldwide, leading to approximately 600,000 deaths each year [1,2]. In the recent decades, therapies for patients with colorectal cancer, including surgical resection, chemotherapy, and radiotherapy, have improved rapidly. However, many patients show a low survival rate because they are diagnosed at an advanced stage [3,4]. Thus, investigation of effective biomarkers is important for diagnosing and treating patients with colorectal cancer.

Long non-coding RNAs (lncRNAs) are 200 nucleotides long non-coding RNAs, which play important roles in cellular growth and apoptosis to modulate cancer pathogenesis and development [5,6]. Notably, evidences indicate that lncRNAs regulate microRNA (miRNA) expression and affect the occurrence and development of a variety of cancers [7–9]. Recently, lncRNA LINC00958 has been found to be involved in the pathogenesis of different cancers. LINC00958 regulates bladder cancer by inhibiting miR-378a-3p and upregulating insulin-like growth factor 1 receptor (IGF1R) [10]. LINC00958 facilitates cell growth by reducing miRNA-625 and promoting NUAK family SnF1-like kinase-1 (NUAK1) in nasopharyngeal carcinoma [11]. LINC00958 is also known to accelerate cell growth and migration by activating JNK/c-JUN signaling in non-small cell lung cancer [12]. Notably, only one study has indicated that LINC00958 inhibits miR-3619-5p and further enhances cell growth and reduces apoptosis in colorectal cancer [13]. However, whether the LINC00958-miR-3064-5p-LEM Domain Containing 1 (LEMD1)-phosphatidylinositol 3-kinase (PI3K)/protein kinase B (AKT) axis plays any important role in the onset and development of colorectal cancer remains unclear.

Based on the malignant behavior of cancer cells, we investigated the effects of LINC00958/miR-3064-5p/LEMD1 on the viability, proliferation, apoptosis, migration, and invasion of colorectal cancer cells. LINC00958 might promote colorectal cancer progression by repressing the effect of miR-3064-5p on LEMD1 to activate the PI3K/AKT signaling pathway; this may potentially provide therapeutic targets for patients with colorectal cancer.

## Materials and methods

### Patient samples, cells, and cell transfection

Colorectal cancer and normal tissues were collected from 29 patients with colorectal cancer, and informed consent was obtained from our hospital. This study was approved by the Ethics Committee of Tianyou Hospital Affiliated to the Wuhan University of Science and Technology. Table 1 shows patient information. Human colorectal cancer cell lines SW620, HCT116, and HCT8 as well as normal colorectal epithelial cells (FHC) were obtained from ATCC (Manassas, VA, USA), and were cultured in DMEM medium (Gibco, USA) with 10% fetal bovine serum (Gibco, USA) at 37°C in 5% CO_2_. HCT116 and HCT8 cells were transfected with siRNA-LINC00958, miR-3064-5p mimics and inhibitor, SiRNA-LEMD1, and negative control (NC) obtained from GenePharm (Shanghai, China) using Lipofectamine 2000 (Invitrogen, USA) for 48 h.

**Table 1.** Baseline characteristics of patients with CRC

Characteristic	Total (n = 29)	
	n	%
**Age (years)**		
≤65	13	44.8
>65	16	55.2
**Gender**		
Male	17	58.6
Female	12	41.4
**Tumor site**		
Colon	18	62.1
Rectum	11	37.9
**Tumor size (cm)**		
≤5	18	62.1
>5	11	37.9
**Pathological T category**		
T1-T2	7	24.1
T3-T4	22	75.9
**Lymph node metastasis**		
M0	15	51.7
M1	14	48.3
**TNM stage**		
I-ll	20	69.0
I–II	9	31.0
**Chemotherapy regimens**		
Well	4	13.8
Moderate	21	72.4
Poor	4	13.8

### Reverse transcription quantitative polymerase chain reaction (RT-qPCR)

Trizol (Cat#: 15596018, Thermo, USA) was used to obtain LINC00958 and LEMD1 mRNA. SuperScript IV One-Step RT-PCR System with ezDNase (12595100, Invitrogen, USA) was used for both RT-qPCR steps, viz. cDNA synthesis and PCR amplification.

PureLink miRNA Isolation Kit (K157001, Invitrogen, USA) was used to obtain miR-3064-5p. miRcute miRNA First Strand cDNA Synthesis kit (KR211, Tiangen, China) was used for cDNA synthesis and miRcute enhanced miRNA fluorescence quantitative detection kit (FP411, Tiangen, China) was used for RT-qPCR carried out on the ABI PRISM 7500 real-time PCR System (Applied Biosystems, USA).

The level of LINC00958 in the nuclear and cytosolic fractions obtained using the PARIS Kit (AM1921, Life, USA) was separately confirmed by RT-qPCR.

Glyceraldehyde-3-phosphate dehydrogenase (GAPDH) was used as an internal control for LEMD1 and cytoplasm control, and small nuclear RNA 6 (U6) was used as an internal control for miR-3064-5p and nuclear control; the analysis was carried out using the 2^−ΔΔCt^ method [14]. Primer sequences are listed in Table 2.

**Table 2.** The sequences of the primers in this study

Primer	Sequences
LINC00958	Forward: 5′-GTCTCCCTGGTTTCTCACAGTT-3′
	Reverse: 5′-TCCCTGGCTACAAATAACCACA-3′
LEMD1	Forward: 5′-AGCTTGGATTTTCACCTGGCCC-3′
	Reverse: 5′-ACAGGTGGTGCACAGGGAGGT-3′
GAPDH	Forward: 5ʹ-CTGGGCTACACTGAGCACC-3’
	Reverse: 5ʹ-AAGTGGTCGTTGAGGGCAATG-3’
U6	Forward: 5ʹ-ACCGTCAGCGAATCCTCTTC-3’
	Reverse: 5ʹ-AACAGGCTCGTGAAAGACCG-3’

### Cell counting kit (CCK-8) assay

Cell viability was detected using CCK-8 kit (Cat#: K1018; APExBIO, China). Transfected HCT116 and HCT8 cells (5 × 10^3^) were seeded in 96-well plates. At 0, 24, 48, 72, and 96 h, 10 µL CCK-8 solution was added to the plate at 37°C followed by 2 h incubation. Then, the optical density (OD) at 450 nm was obtained using a multimode plate reader (Biotek, USA) [15].

### 5-bromo-2′-deoxyuridine (BrdU) assay

Cell proliferation was measured using a BrdU cell proliferation kit (ab142567, Abcam, UK). Approximately 1 × 10^4^ transfected HCT116 and HCT8 cells were cultured in 96-well plates for 48 h. Afterward, the medium was replaced with DMEM medium, and 10 μL BrdU solution was added to the plate. After 4 h of incubation, the cells were washed twice and incubated with secondary antibody for 1 h. OD_450 nm_ was measured using a microplate reader (Biotek, USA) [16].

### Apoptosis assay

Cell apoptosis was determined using the Annexin V-fluorescein isothiocyanate (FITC)-propidium iodide (PI) apoptosis detection kit (Cat#: 556547; BD, USA). Transfected HCT116 and HCT8 cells were harvested, washed twice, and suspended in binding buffer. After 20 min incubation with 5 µL FITC and 10 µL PI in the dark at 4°C, cells were washed twice and suspended in binding buffer and subjected to flow cytometry (BD, USA) using the Cell-Quest software (BD) [17].

### Cell migration assay

Cell migration was analyzed by wound healing assay as described previously [18]. Transfected HCT116 and HCT8 cells (1 × 10^6^) were cultured in 6-well plates. When cells reached 80% confluence, a 200 μL pipette tip was used to draw a straight line in the middle of each cell monolayer, and cells were washed twice to remove the detached cells. Images of the scratch at 0 and 24 h were captured using a light microscope.

### Cell invasion assay

A transwell chamber with 8-µm pores (Cat#: #3244, Coring, USA) was prepared with matrigel (Corning, USA). A 10% FBS cell culture medium was added to the basolateral chambers. Transfected HCT116 and HCT8 cells (8 × 10^4^) were seeded into the apical chamber. After 48 h, the invaded cells were fixed with 4% paraformaldehyde and stained with 0.1% crystal violet. Finally, images were captured using a BX43 light microscope (Olympus, Japan), and the number of invasive cells was counted [19].

### Dual luciferase reporter assay

The assay was performed as described previously [20]. The LINC00958 mutated (MUT) sequences and LEMD1 3ʹ-UTR mutated sequences were synthesized using QuikChange XL Site-Directed Mutagenesis Kit (Stratagene, USA). Then, LINC00958 wild-type (WT) or MUT sequences and the LEMD1 3ʹ-UTR WT or MUT sequences were cloned in the psiCHECK2 vector (GenePharm, Shanghai, China). The miR-3064-5p mimic or mimic-NC was transfected into HCT116 and HCT8 cells with either of the psiCHECK2 vector for 48 h. Luciferase activities of firefly and Renilla were measured using the Dual-Luciferase Reporter Assay System (Cat#: E1910, Promega, USA). Renilla luciferase activity was used as the control.

### RNA immunoprecipitation (RIP) assay

The interaction of LINC00958 and miR-3064-5p in HCT116 and HCT8 cells was determined using the RIP kit (Cat#: P0101, Geneseed, China). Cell lysates from cells treated with miR-3064-5p mimic or NC were incubated with anti-immunoglobulin G (IgG) or anti-Argonaute 2 (AGO2). After 2 h, magnetic beads were added to the solution at 4°C. The next day, LINC00958 was purified and analyzed by RT-qPCR [21].

### RNA-pull down detection

The HCT116 and HCT8 cells were tagged with biotin-labeled miR-3064-5p (Bio-miR-3064-5p) or Bio-miR-NC (Thermo, USA) for 48 h. Then, the cell lysates were added to the beads (Cat#: S1420S, NEB, UK) at 4°C on a rotator and incubated overnight. The enriched RNAs were purified using the PureYield RNA Midiprep System (Promega, USA). RT-qPCR was performed to measure LEMD1 enrichment [22].

### Western blot analysis

Lysates of transfected HCT116 and HCT8 cells were obtained using RIPA buffer (Cat#: #20-188, Sigma, USA). Protein samples were separated using 10% sodium dodecyl sulfate polyacrylamide gel electrophoresis (SDS-PAGE) and then transferred to polyvinylidene (PVDF) membranes; the membranes were blocked using 5% nonfat milk for 1 h. After washing twice, the membranes were incubated with anti-LEMD1 (1:1000, Cat#: ab201206, Abcam, UK), PI3K (1:1000, Cat#: ab86714, Abcam), p-PI3K (1:750, Cat#: ab182651. Abcam), AKT (1:1000, Cat#: 4691, Cell Signaling Technology, USA), p-AKT (1:2000, Cat#: 4060; 1:2000, Cell Signaling Technology), and anti-GAPDH (1:2000, Cat#: 5174, CST, USA) overnight at 4°C. The membranes were then washed twice and were incubated with horse radish peroxidase (HRP)-linked anti-rabbit antibody (1:5000, Cat#: ab6721, Abcam, UK) for 1 h. Finally, protein bands were developed using the EasyBlot ECL kit (#C506668; Sangon, China), and band densities were analyzed using Image J software (National Institute of Health, USA). Relative LEMD1 protein level was normalized to that of GAPDH [23].

### Statistical analysis

Data were analyzed using GraphPad Prism 8.0 (GraphPad Prism, USA) and presented as the mean ± standard deviation obtained from triplicate experiments. Comparisons between two and more groups were evaluated using Student’s t-test or analysis of variance (ANOVA), respectively. Pearson correlation analysis was performed for analyzing the associations between LINC00958 and miR-3064-5p expression, and LEMD1 and miR-3064-5p expression. Differences were considered significant at *P* < 0.05.

## Results

To begin with, we identified miR-3064-5p and LEMD1 as genes of interest through bioinformatics analysis. In addition, the expression level of LINC00958 was found to have increased in colorectal cancer. Biological function analysis showed that knockdown of LINC00958 inhibited the viability, proliferation, migration, and invasion of cancer cells and promoted cell apoptosis. Targeting analysis showed that LINC00958 affected tumor progression by negatively regulating miR-3064-5p. Finally, we demonstrated that miR-3064-5p controls the phosphorylation of PI3K and AKT by binding to the classical transcription factor LEMD1. Thus, the LINC00958/miR-3064-5p/LEMD1/PI3K/AKT axis was confirmed to influence the progression of colorectal cancer.

### LEMD1 was significantly upregulated and miR-3064-5p was downregulated in colorectal cancer

**Figure 1.**
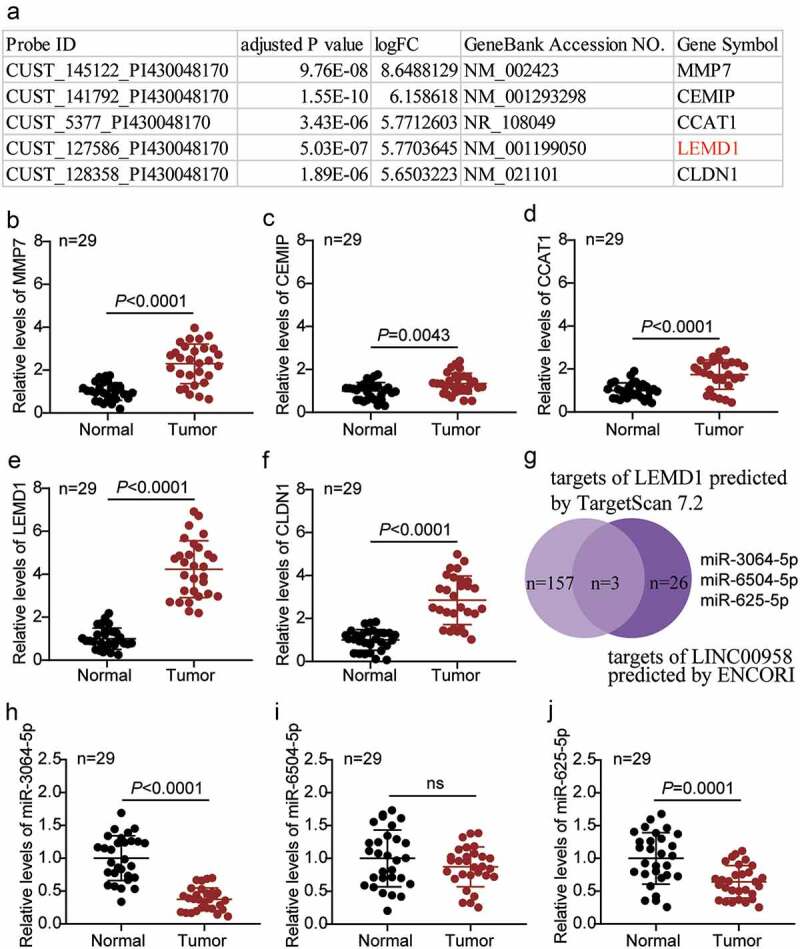
LEMD1 was found significantly upregulated and miR-3064-5p was significant downregulated in colorectal cancer

The top five most upregulated genes in colorectal cancer in the GSE126095 data series are listed in Figure 1a. The expression of five genes, namely MMP7, CEMIP, CCAT1, LEMD1, and CLDN1, was also detected in the tissue samples collected in this study. We found that LEMD1 was the most significantly upregulated gene in our samples (Figure 1b-f). The expression of LEMD1 in cancer tissues was almost fourfold higher than that in the adjacent healthy tissues (Figure 1e). We then analyzed the possible target miRNAs of LEMD1 using the TargetScan Human 7.2 algorithm and the potential target miRNAs of LINC00958 using the ENCORI algorithm. miR-3064-5p, miR-6504-5p, and miR-625-5p were identified as common targets of LINC00958 and *LEMD1* mRNA (Figure 1g). Among the three miRNAs, miR-3064-5p was the most downregulated in the cancer tissue samples collected in our study (Figure 1h-j).

(a) The top five most upregulated genes from GSE126095 data series. FC: fold change. (b) The expression of MMP7 in the collected tissue samples. (c) The expression of CEMIP in the collected tissue samples. (d) The expression of CCAT1 in the collected tissue samples. (e) The expression of LEMD1 in the collected tissue samples. (f) The expression of CLDN1 in the collected tissue samples. (g-j) The identification of miR-3064-5p, the most significantly downregulated miRNA in our collected CRC samples as the interested miRNA.

### LINC00958 facilitated colorectal cancer cells progression

**Figure 2.**
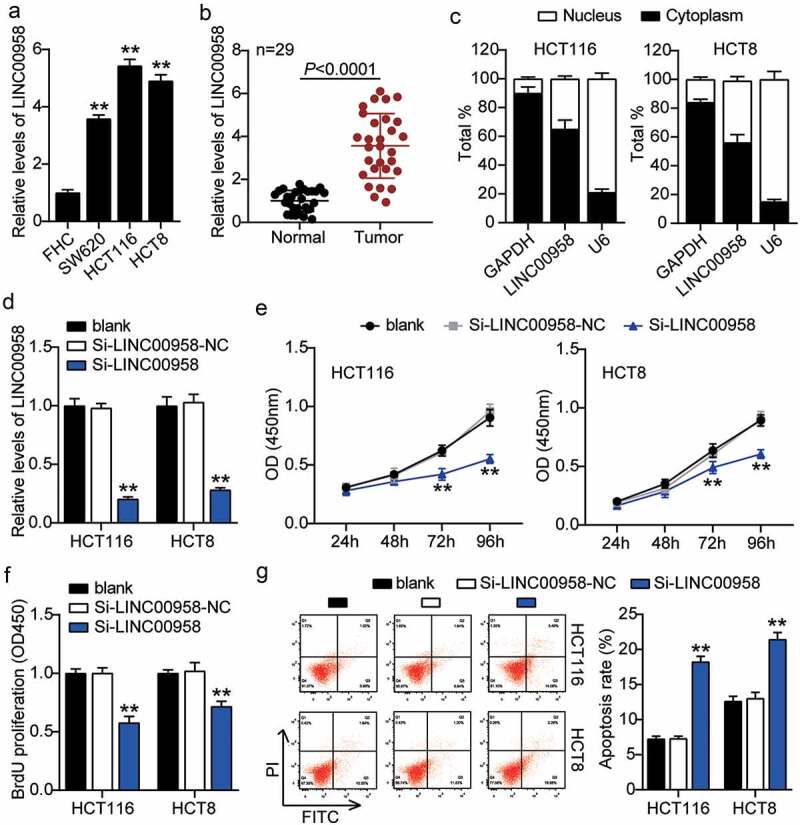
LINC00958 promoted cell growth, but inhibited cell apoptosis of colorectal cancer cells

**Figure 3.**
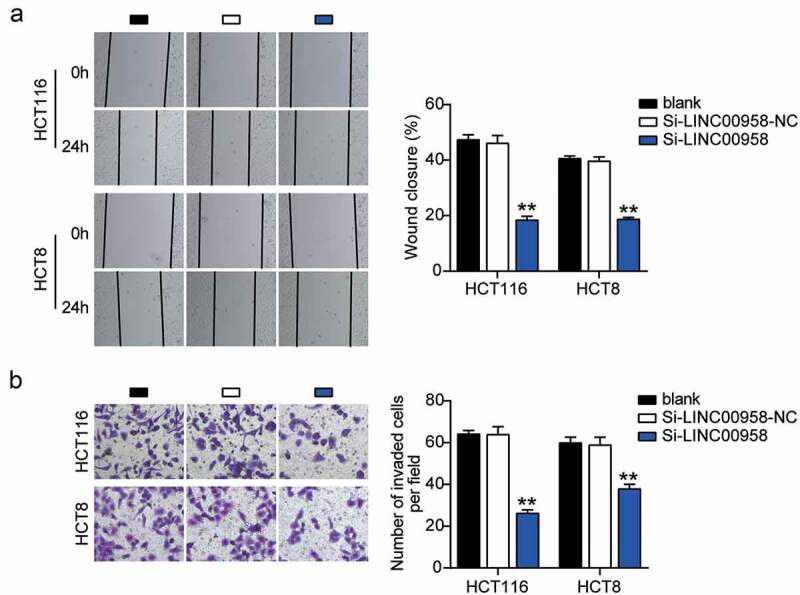
LINC00958 promoted cell migration and invasion of colorectal cancer cells

First, we determined the levels of LINC00958 in colorectal cancer cells and tissues. As the data show, colorectal cancer cells showed dramatically higher LINC00958 expression than that observed in normal FHC cells; this was especially true for HCT116 and HCT8 cells, which were chosen for subsequent studies. At the same time, colorectal cancer tissues also displayed enhanced LINC00958 expression compared to the normal tissues (Figure 2a,b). Next, we confirmed that LINC00958 mainly existed in the cytoplasm of both HCT116 and HCT8 cells (Figure 2c). After transfecting siRNA-LINC00958 and negative control (NC) into HCT116 and HCT8 cells, the si-LINC00958 was downregulated by approximately 70% when compared with control cells, in both cell lines (Figure 2d). CCK-8 assay showed that si-LINC00958 groups significantly inhibited cell viability in both cell lines when compared to the control cells (Figure 2e). The si-LINC00958 groups also showed approximately 30% decrease in cell proliferation compared with control cells in both cell lines (Figure 2f). Furthermore, the si-LINC00958 groups showed nearly twofold cell apoptosis compared with control cells in both cell lines (Figure 2g). On the other hand, si-LINC00958 groups showed approximately half of the cell migration and invasion compared to that observed for the control cells, in both cell types (Figures 3a,b). These results indicated that LINC00958 facilitated the progression of colorectal cancer.

(a) Measurement of LINC00958 expression in colorectal cancer cell lines (SW620, HCT116 and HCT8) and normal colorectal epithelial cells FHC. (b) RT-qPCR analysis of LINC00958 in colorectal cancer tissues and normal tissues. (c) The existence of GAPDH, LINC00958, and U6 in the cytoplasm and nucleus were detected in HCT116 and HCT8 cell lines by RT-qPCR. (d) RT-qPCR analysis of LINC00958 in HCT116 and HCT8 cells transfected with NC and si-LINC00958. (e) Cell viability was detected in HCT116 and HCT8 cells transfected with NC and si-LINC00958 by CCK8 assay. (f) Cell proliferation was detected in HCT116 and HCT8 cells transfected with NC and si-LINC00958 by EdU assay. (g) Cell apoptosis was determined in HCT116 and HCT8 cells transfected with NC and si-LINC00958 by FITC apoptosis detection kit. *, *P* < 0.05; **, *P* < 0.001 compared with blank. NC, negative control; si-LINC00958, siRNA-LINC00958.

(a) Cell migration ability was determined in HCT116 and HCT8 cells transfected with NC and si-LINC00958 by wound healing assay. (b) Cell invasion was determined in HCT116 and HCT8 cells transfected with NC and si-LINC00958 by transwell assay. *, *P* < 0.05; **, *P* < 0.001 compared with blank. NC, negative control; si-LINC00958, siRNA-LINC00958.

### LINC00958 sponged miR-3064-5p in colorectal cancer cells

**Figure 4.**
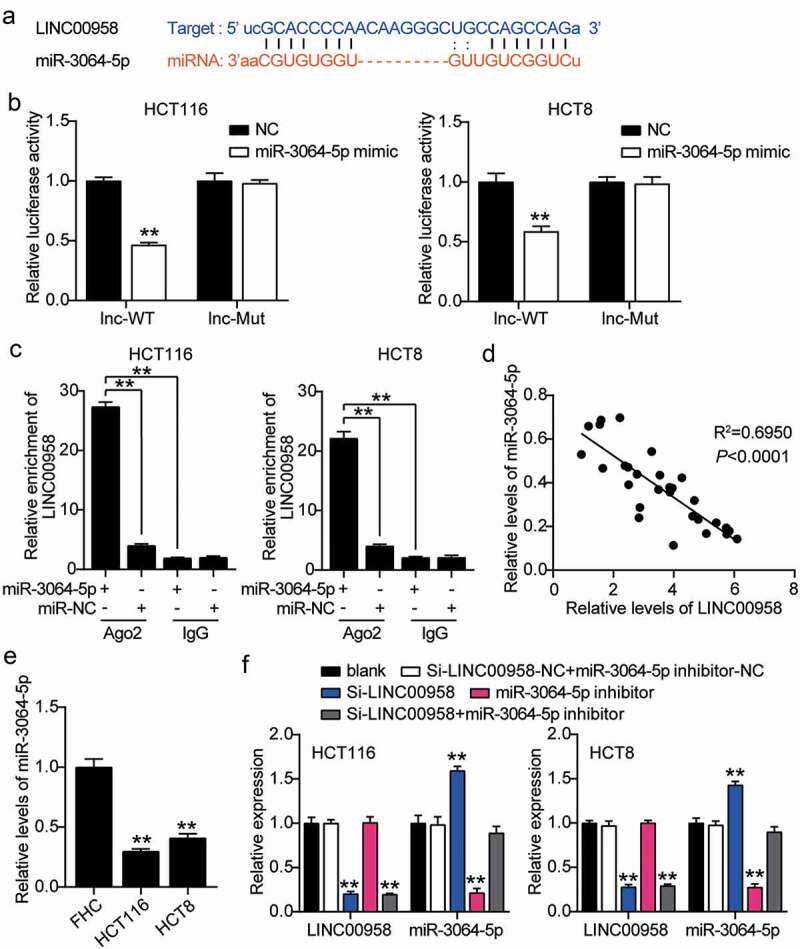
LINC00958 interacted with miR-3064-5p in colorectal cancer cells

The binding sequence between LINC00958 and miR-3064-5p is shown in Figure 4a. miR-3064-5p mimics or NC and psiCHECK2 LINC00958-wide type (WT) vectors or psiCHECK2 LINC00958-Mut vectors were co-transfected into HCT116 and HCT8 cells. The cells treated with miR-3064-5p mimics and psiCHECK2 LINC00958-Mut showed 50% reduction in luciferase activity compared with the NC group (Figure 4b). The interaction between LINC00958 and miR-3064-5p was confirmed by RNA immunoprecipitation (RIP) in both HCT116 and HCT8 cells (Figure 4c). miR-3064-5p levels were negatively correlated with LINC00958 levels in colorectal cancer tissues (Figure 4d). HCT116 and HCT8 cells also showed more than 50% decrease in miR-3064-5p levels compared with normal cells (Figure 4e). Next, we transfected Si-LINC00958, miR-3064-5p inhibitor, and NC into HCT116 and HCT8 cells to determine the role of the LINC00958-miR-3064-5p axis. The si-LINC00958 groups showed a 1.5-fold increase in miR-3064-5p levels, but a 70% reduction in LINC00958 levels compared with control cells. The miR-3064-5p inhibitor groups showed 70% reduction in miR-3064-5p levels but the LINC00958 levels were similar to those observed in the control cells. In addition, the si-LINC00958+ miR-3064-5p inhibitor groups showed a 70% decrease in LINC00958 levels, but the same miR-3064-5p levels compared with control cells (Figure 4f). Collectively, LINC00958 sponged miR-3064-5p and inhibited its expression in colorectal cancer cells.

(a) StarBase analysis showed the predicted binding sequences of miR-3064-5p for LINC00958. (b) Dual luciferase assay was performed in cells co-transfected with plasmids LINC00958-WT or LINC00958-MUT1 or MUT2 and NC mimics or miR-3064-5p mimic in HCT116 and HCT8 cells. (c) The enrichment of LINC00958 by treating with miR-3064-5p mimic in HCT116 and HCT8 cells through RIP assay. (d) Correlation analysis between miR-3064-5p expression and LINC00958 expression. (e) RT-qPCR detection of miR-3064-5p expression in colorectal cancer cells (HCT116 and HCT8 cells) and normal FHC cells. (f) RT-qPCR detection of miR-3064-5p and LINC00958 expression in HCT116 and HCT8 cells transfected with NC, Si-LINC00958, miR-3064-5p inhibitor, and Si-LINC00958+ miR-3064-5p inhibitor. *, *P* < 0.05; **, *P* < 0.001 compared with blank. ^#^, *P* < 0.05; ^##^, *P* < 0.001 compared with Si-LINC00958+ miR-3064-5p inhibitor. NC, negative control; WT, wild-type; MUT, Mutant; Si-LINC00958, SiRNA-LINC00958; Si-LINC00958+ miR-3064-5p inhibitor, SiRNA-LINC00958+ miR-3064-5p inhibitor.

### Sponging miR-3064-5p by LINC00958 facilitated the development of colorectal cancer cells

**Figure 5.**
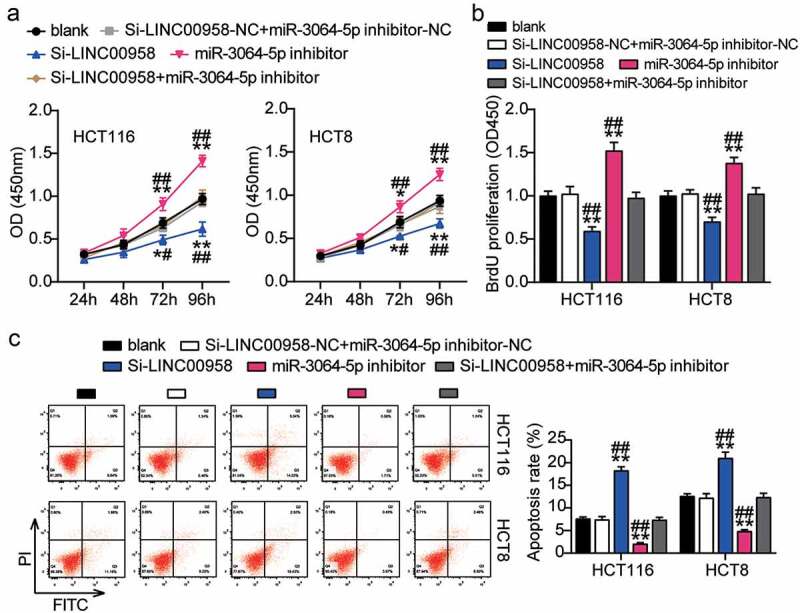
LINC00958 sponging miR-3064-5p facilitated cell proliferation and repressed cell apoptosis of colorectal cancer cells

**Figure 6.**
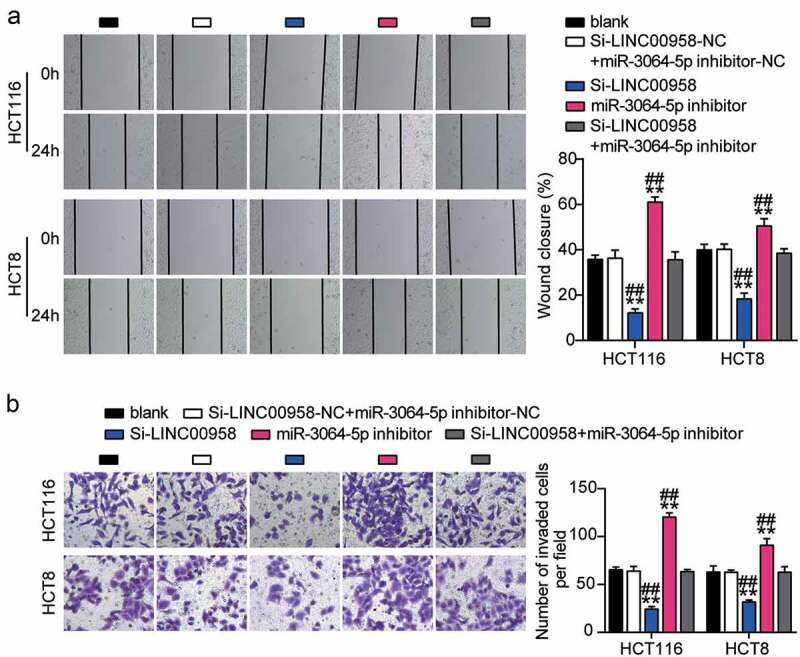
LINC00958 sponging miR-3064-5p enhanced cell migration and invasion of colorectal cancer cells

Next, we assessed the function of the LINC00958-miR-3064-5p axis in colorectal cancer and found that miR-3064-5p inhibitor groups showed significantly enhanced cell viability compared with the control cells (Figure 5a). Consistently, miR-3064-5p inhibitor groups were observed to promote the cell proliferation 1.5-fold higher than that observed for the control cells (Figure 5b). However, miR-3064-5p inhibitor groups showed decreased cell apoptosis compared with the control cells (Figure 5c). On the other hand, these groups showed approximately twofold enhanced cell migration and 1.3-fold enhanced cell invasion when compared with the control cells in HCT116 and HCT8 cells, respectively (Figure 6a,b). It was further found that si-LINC00958 significantly inhibited the effect of miR-3064-5p inhibitor in both HCT116 and HCT8 cells.

(a) Cell viability was detected in HCT116 and HCT8 cells transfected with NC, Si-LINC00958, miR-3064-5p inhibitor, and Si-LINC00958+ miR-3064-5p inhibitor. (b) Cell proliferation was detected in HCT116 and HCT8 cells transfected with NC, Si-LINC00958, miR-3064-5p inhibitor, and Si-LINC00958+ miR-3064-5p inhibitor. (c) Cell apoptosis was detected in HCT116 and HCT8 cells transfected with NC, Si-LINC00958, miR-3064-5p inhibitor, and Si-LINC00958+ miR-3064-5p inhibitor. *, *P* < 0.05; **, *P* < 0.001 compared with blank. ^#^, *P* < 0.05; ^##^, *P* < 0.001 compared with Si-LINC00958+ miR-3064-5p inhibitor. NC, negative control; Si-LINC00958, SiRNA-LINC00958; Si-LINC00958+ miR-3064-5p inhibitor, SiRNA-LINC00958+ miR-3064-5p inhibitor.

(a) Cell migration was detected in HCT116 and HCT8 cells transfected with NC, Si-LINC00958, miR-3064-5p inhibitor, and Si-LINC00958+ miR-3064-5p inhibitor. (b) Cell invasion was detected in HCT116 and HCT8 cells transfected with NC, Si-LINC00958, miR-3064-5p inhibitor, and Si-LINC00958+ miR-3064-5p inhibitor. *, *P* < 0.05; **, *P* < 0.001 compared with blank. ^#^, *P* < 0.05; ^##^, *P* < 0.001 compared with Si-LINC00958+ miR-3064-5p inhibitor. NC, negative control; Si-LINC00958, SiRNA-LINC00958; Si-LINC00958+ miR-3064-5p inhibitor, SiRNA-LINC00958+ miR-3064-5p inhibitor.

### LEMD1 was targeted by miR-3064-5p in colorectal cancer cells

**Figure 7.**
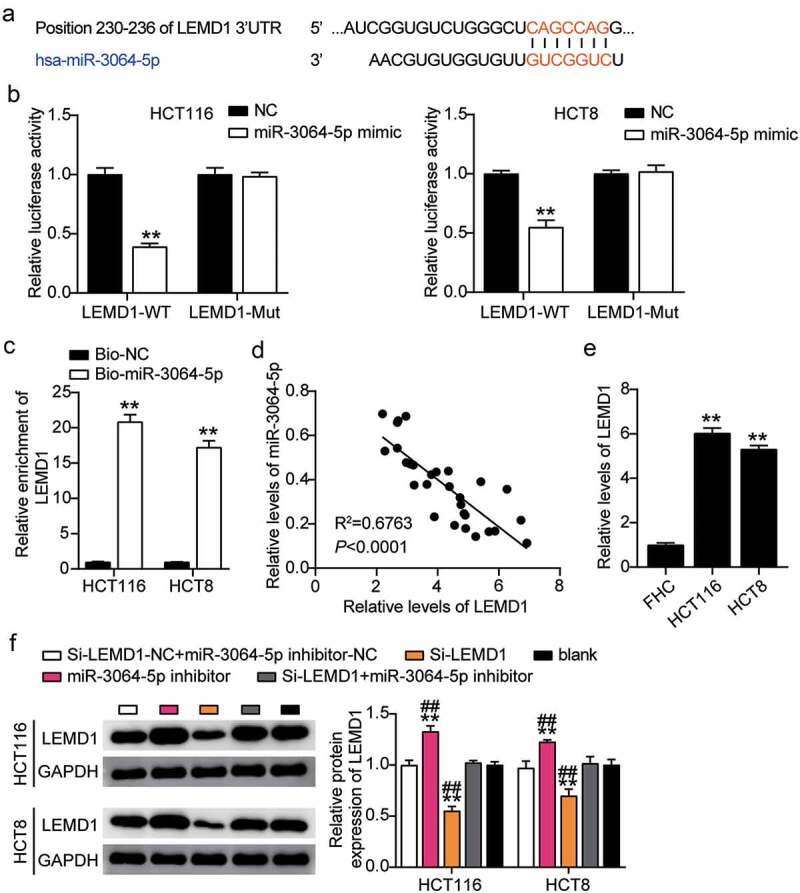
MiR-3064-5p targeted to LEMD1 and inhibited the expression of LEMD1 in colorectal cancer cells

The binding site between LEMD1 and miR-3064-5p was obtained from starBase (Figure 7a). HCT116 and HCT8 cells co-transfected with miR-3064-5p mimics and psiCHECK2 LEMD1 3′-UTR WT vectors showed nearly 50% decreased luciferase activity; however, this was not observed for the psiCHECK2 LEMD1 3′-UTR Mut vectors, suggesting an interaction between miR-3064-5p and LEMD1 (Figure 7b). The interaction between miR-3064-5p and LEMD1 was verified by an RNA pull-down assay in both the cell lines (Figure 7c). A negative correlation between miR-3064-5p and LEMD1 was found in colorectal cancer tissues (Figure 7d). The expression of LEMD1 in HCT116 and HCT8 cells was found to have been significantly elevated by approximately sixfold compared to that in the normal cells (Figure 7e). To determine the effect of miR-3064-5p-LEMD1 axis in colorectal cancer, we transfected siRNA-LEMD1, miR-3064-5p inhibitor, and NC into HCT116 and HCT8 cells. The si-LEMD1 groups showed approximately 50% downregulation in LEMD1 protein levels, whereas those in the miR-3064-5p inhibitor groups were promoted by 1.3-fold when compared with the control cells (Figure 7f).

(a) StarBase showed the predicted binding sequences of LEMD1 and miR-3064-5p. (b) Dual luciferase assay was performed in cells co-transfected with plasmids LEMD1 3ʹUTR-WT or LEMD1 3ʹUTR-MUT and NC mimics or miR-3064-5p mimic in HCT116 and HCT8 cells. (c) RNA pull-down assay was performed between miR-3064-5p and LEMD1 in HCT116 and HCT8 cells. (d) The correlation between the relative expression level of miR-3064-5p and LEMD1 in colorectal cancer tissues. (e) RT-qPCR detection of LEMD1 expression in colorectal cancer cells (HCT116 and HCT8 cells) and normal FHC cells. (f) Measurement of LEMD1 protein expression in HCT116 and HCT8 cells transfected with NC, Si-LEMD1, miR-3064-5p inhibitor, and Si-LEMD1+ miR-3064-5p inhibitor. *, *P* < 0.05; **, *P* < 0.001 compared with blank. ^#^, *P* < 0.05; ^##^, *P* < 0.001 compared with Si-LEMD1+ inhibitor. NC, negative control; WT, wild-type; MUT, Mutant; Si-LEMD1, SiRNA-LEMD1; Si-LEMD1+ miR-3064-5p inhibitor, SiRNA-LEMD1+ miR-3064-5p inhibitor.

### miR-3064-5p attenuated colorectal cancer cell progression by inhibiting LEMD1

**Figure 8.**
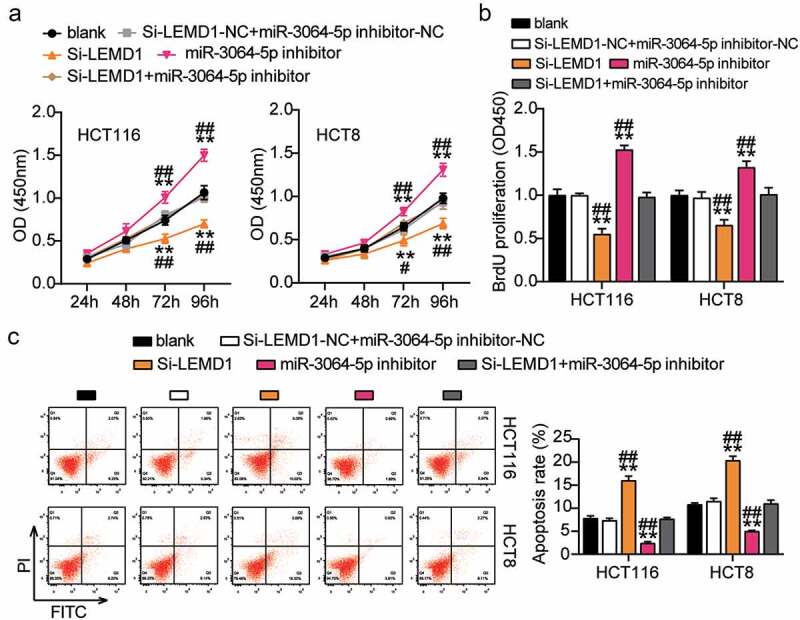
MiR-3064-5p attenuated cell proliferation and elevated cell apoptosis by inhibiting LEMD1

**Figure 9.**
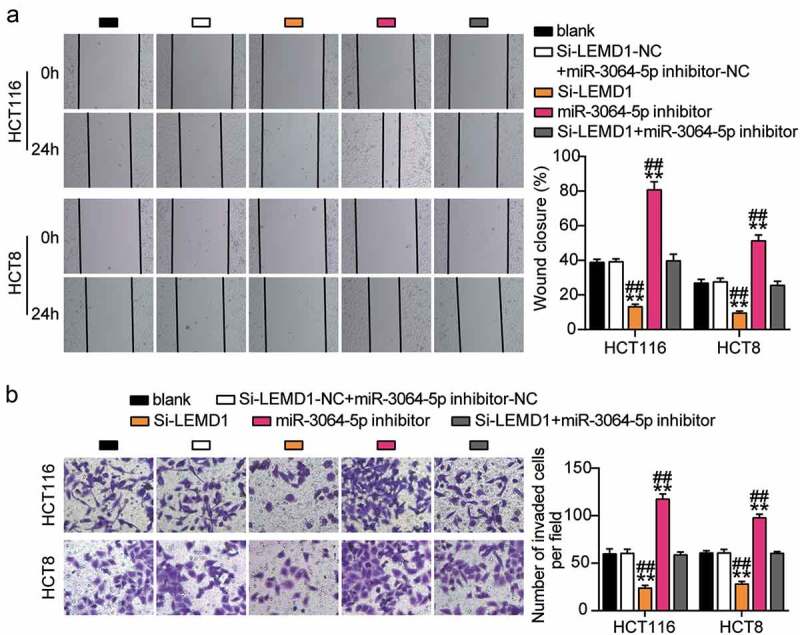
MiR-3064-5p repressed cell migration and invasion by inhibiting LEMD1

The si-LEMD1 groups showed significant inhibition of cell viability when compared with the control cells (Figure 8a). The si-LEMD1 groups showed nearly 50% repression of cell proliferation when compared with the control cells (Figure 8b). However, the si-LEMD1 groups showed an approximately twofold increase in cell apoptosis compared with the control cells (Figure 8c). Additionally, we found that the si-LEMD1 groups showed nearly 50% reduction in cell migration and invasion compared with the control cells (Figures 9a,b). However, these effects were abrogated by si-LEMD1+ miR-3064-5p inhibitor treatment in both HCT116 and HCT8 cells. Thus, miR-3064-5p was observed to attenuate colorectal cancer cell progression by inhibiting LEMD1 expression.

(a) Cell viability was detected in HCT116 and HCT8 cells transfected with NC, Si-LEMD1, miR-3064-5p inhibitor, and Si-LEMD1+ miR-3064-5p inhibitor. (b) Cell proliferation was detected in HCT116 and HCT8 cells transfected with NC, Si-LEMD1, miR-3064-5p inhibitor, and Si-LEMD1+ miR-3064-5p inhibitor. (c) Cell apoptosis was detected in HCT116 and HCT8 cells transfected with NC, Si-LEMD1, miR-3064-5p inhibitor, and Si-LEMD1+ miR-3064-5p inhibitor. *, *P* < 0.05; **, *P* < 0.001 compared with blank. ^#^, *P* < 0.05; ^##^, *P* < 0.001 compared with Si-LEMD1+ miR-3064-5p inhibitor. NC, negative control; Si-LEMD1, SiRNA-LEMD1; Si-LEMD1+ miR-3064-5p inhibitor, SiRNA-LEMD1+ miR-3064-5p inhibitor.

(a) Cell migration was detected in HCT116 and HCT8 cells transfected with NC, Si-LEMD1, miR-3064-5p inhibitor, and Si-LEMD1+ miR-3064-5p inhibitor. (b) Cell invasion was detected in HCT116 and HCT8 cells transfected with NC, Si-LEMD1, miR-3064-5p inhibitor, and Si-LEMD1+ miR-3064-5p inhibitor. *, *P* < 0.05; **, *P* < 0.001 compared with blank. ^#^, *P* < 0.05; ^##^, *P* < 0.001 compared with Si-LEMD1+ inhibitor. NC, negative control; Si-LEMD1, SiRNA-LEMD1; Si-LEMD1+ miR-3064-5p inhibitor, SiRNA-LEMD1+ miR-3064-5p inhibitor.

### miR-3064-5p was found to regulate the downstream PI3K-AKT signaling pathway through LEMD1

**Figure 10.**
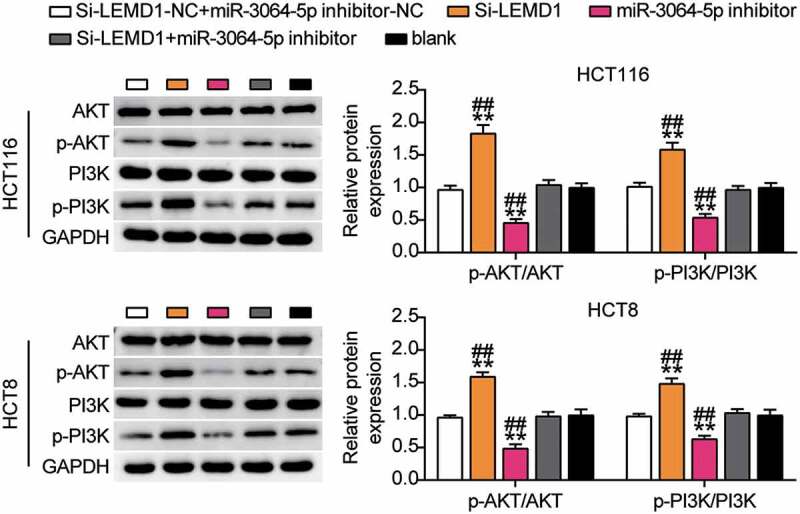
MiR-3064-5p regulates the downstream PI3K-AKT signaling pathway through LEMD1

Furthermore, we studied the expression levels of PI3K-AKT signaling pathway-related proteins. Western blotting showed that p-PI3K and p-AKT protein levels in the si-LEMD1 group were significantly lower than those in the control group, while p-PI3K and p-AKT protein levels were elevated in the miR-3064-5p inhibitor group. In addition, silencing LEMD1 reversed the effects of miR-3064-5p interference on the levels of p-PI3K and p-AKT (Figure 10). Considering the targeted regulation of LEMD1 by miR-3064-5p, we hypothesized that interference with miR-3064-5p could activate the downstream PI3K-AKT signaling pathway via LEMD1.

**, *P* < 0.001 compared with blank. ^##^, *P* < 0.001 compared with Si-LEMD1+ inhibitor. NC, negative control; Si-LEMD1, SiRNA-LEMD1; Si-LEMD1+ miR-3064-5p inhibitor, SiRNA-LEMD1+ miR-3064-5p inhibitor.

## Discussion

The current study showed that LINC00958 inhibited miR-3064-5p expression and promoted the expression of LEMD1, which effectively enhanced cell growth and arrested apoptosis of colorectal cancer cells.

Accumulating evidence suggests that LINC00958 plays an important role in cancer progression [24–26]. Wang et al. reported that LINC00958 accelerated cell growth and metastasis by reducing miR-625-5p to elevate LRRC8E expression in cervical cancer [24]. Chen et al. have revealed that LINC00958 inhibited miR-627-5p and upregulated YBX2 to enhance oral squamous cell carcinoma cell growth [25]. Another study indicated that LINC00958 accelerated tumor initiation in pancreatic cancer by downregulating miRNA-330-5p but upregulating PAX8 [26]. Regarding colorectal cancer, only one study has demonstrated that LINC00958 significantly promoted cell growth by inhibiting miR-3619-5p expression. Downregulation of LINC00958 reversed tumorigenesis by increasing miR-3619-5p expression [13]. Our study showed that LINC00958 levels in colorectal cancer tissues and cells were evidently increased. LINC00958 silencing attenuated cell growth and boosted apoptosis in HCT116 and HCT8 cells. Additionally, LINC00958 sponged miR-3064-5p, which suppressed the role of miR-3064-5p in preventing colorectal cancer progression.

miR-3064-5p has been previously reported to participate in the pathogenesis of cancers [27–29]. For instance, CircPRRX1 was shown to promote doxorubicin resistance in gastric cancer by inhibiting miR-3064-5p and enhancing protein tyrosine phosphatase, non-receptor type 14 (PTPN14) signaling [27]. In addition, lncRNA PXN-AS1 repressed cell growth by reducing miR-3064-5p and upregulating phosphatidylinositol-5-phosphate 4-kinase type 2 beta (PIP4K2B) expression in pancreatic cancer [28]. Furthermore, miRNA-3064-5p dramatically hampered the cell growth and invasion by suppressing telomerase reverse transcriptase in ovarian cancer [29]. Notably, the role of miR-3064-5p in colorectal cancer has remained unknown. We demonstrated that miR-3064-5p expression in colorectal cancer tissues and cells was reduced. Notably, miR-3064-5p was negatively correlated with LINC00958 or LEMD1 expression in colorectal cancer tissues. miR-3064-5p inhibitor accelerated cell growth but hampered cell apoptosis; however, LINC00958 reduced miR-3064-5p expression to elevate the progression of colorectal cancer.

Previous studies have identified *LEMD1* as an oncogene in various cancers [30–32]. For instance, LEMD1 promotes cell growth, invasion, and endothelial transmigration in oral squamous cell carcinoma (OSCC) [30]. Additionally, they found that the sushi repeat-containing protein X-linked 2 (SRPX2) is a downstream signal of LEMD1, which acts as a tumor genesis gene in OSCC [31]. Overexpression of LEMD1 was also observed during the initiation of colorectal cancer, and downregulation of LEMD1 significantly destroyed the maintenance of colorectal cancer [33,34]. In our study, LEMD1 expression in colorectal cancer tissues and cells was found to have been significantly increased. The silencing of *LEMD1* dramatically hampered cell growth and enhanced apoptosis in colorectal cancer cells. Notably, we observed that *LEMD1* expression was attenuated by the LINC00958-miR-3064-5p axis in colorectal cancer.

Abnormal activation of PI3K, a member of the 3ʹ-hydroxy phosphorylated conserved lipokinase family of phosphatidylinositol, is commonly observed in human cancers [35]. This protein phosphorylates PIP2 to produce PIP3 by regulating the extracellular protein kinase levels [36]. PIP3 is an important second messenger involved in AKT recruitment and activation of the cell survival signal response [37]. A previous study found that LEMD1 regulates the proliferation and apoptosis of gastric cancer cells by activating the PI3K/AKT signaling pathway [32]. Furthermore, phosphorylation of PI3K and AKT has been shown to support the tumor environment in non-small cell lung carcinoma and colorectal cancer [38,39]. Similar to previous studies, this study found that knocking down *LEMD1* inhibited the phosphorylation of PI3K and AKT. In addition, rescue experiments showed that low expression of LEMD1 reversed the inhibitory effect of miR-3064-5p interference on the PI3K/AKT signaling pathway.

Nevertheless, this study has several limitations. The absence of animal studies has resulted in insufficient evidence to support the effect of LINC00958 on the growth of colorectal cancer cells in vivo. In addition, the correlation between LINC00958/miR-3064-5p/LEMD1, patient prognosis, and histology needs to be further investigated. We have only examined the effects of LINC00958, miR-3064-5p, and LEMD1 on the activity, proliferation, migration, invasion, and apoptosis of HCT116 and HCT8 cells based on the colorectal cancer cell level. However, the therapeutic effect of LINC00958/miR-3064-5p/LEMD1, such as exploring the correlation with prognosis, has not been studied at the clinical level, which will be explored in the future studies to provide an effective biomarker for the treatment of colorectal cancer.

## Conclusion

Our study showed that LINC00958 and LEMD1 were upregulated and miR-3064-5p was downregulated in colorectal cancer. In summary, we uncovered for the first time that LINC00958 sponging miR-3064-5p promotes the viability, proliferation, migration, and invasion of colorectal cancer cells and inhibits apoptosis by elevating LEMD1 expression and activating the PI3K/AKT signaling pathway. This may provide effective biomarkers based on the LINC00958-miR-3064-5p-LEMD1 axis for the treatment of colorectal cancer.

## Supplementary Material

Supplemental Material

## Data Availability

The datasets used and/or analyzed during the current study are available from the corresponding author (yaolei563@163.com) on reasonable request.
