# Supplementary material for: Long non-coding RNA LINC00958 promotes colorectal cancer progression by enhancing the expression of LEM domain containing 1 via microRNA miR-3064-5p
Source: Bioengineered. 2021 Oct 21;12(1):8100–15. doi: 10.1080/21655979.2021.1985259 (PMC8806780; doi:10.1080/21655979.2021.1985259)

**LINC00958 promotes colorectal cancer progression by enhancing LEMD1 expression via miR-3064-5p**

*Running title:* LINC00958 promotes colorectal cancer progression

**Zhaoxia Luo^1, #^, Shunxin Hao^1, #^, Jian Yuan^1^, Kai Zhu^1^, Shuo Liu^1^, Jing Zhang^1^, Lei Yao^1, *^**

^1^Department of General Surgery, Tianyou Hospital Affiliated to Wuhan University of Science and Technology, Wuhan 430064, Hubei, China

^*^Corresponding author: **Lei Yao**, Department of General Surgery, Tianyou Hospital Affiliated to Wuhan University of Science and Technology, No. 9 Tujialin, Wuchang District, Wuhan 430064, Hubei, China

Email: yaolei563@163.com

Tel/Fax: +86 027-51228666

^#^These authors contributed equally to this work.

HCT116 LEMD1 for Fig.7F


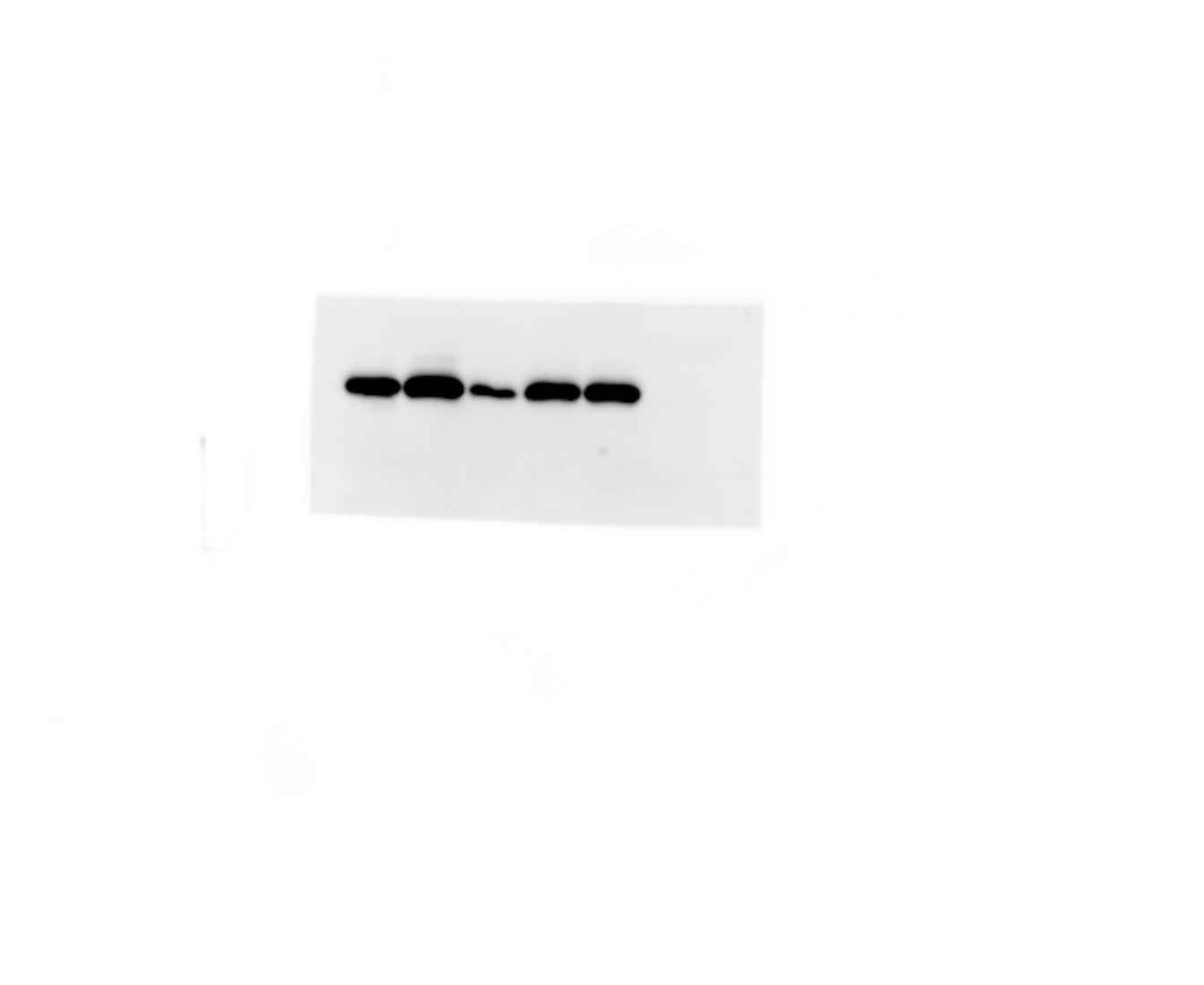


HCT116 GAPDH for Fig.7F


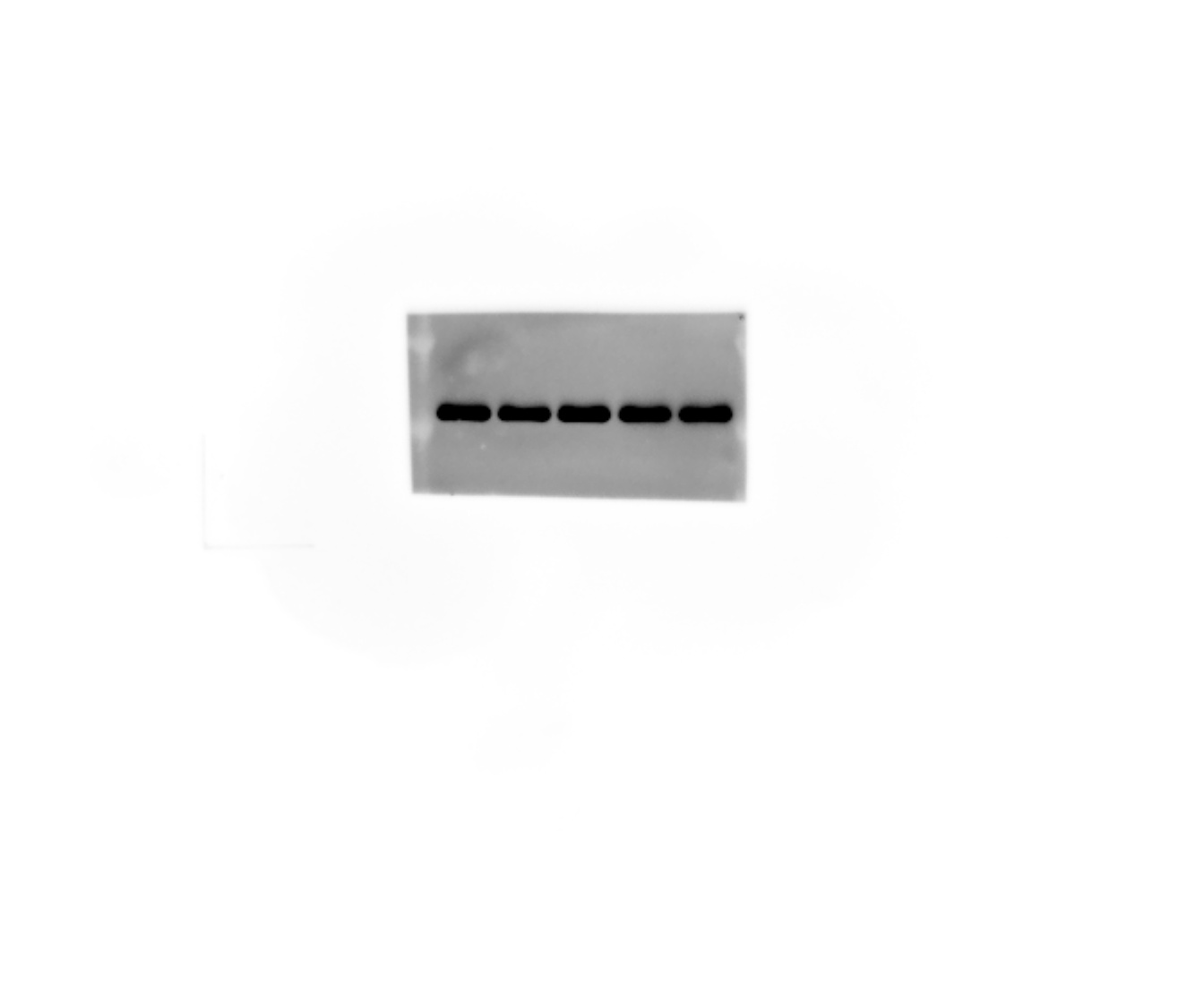


HCT8 LEMD1 for Fig.7F


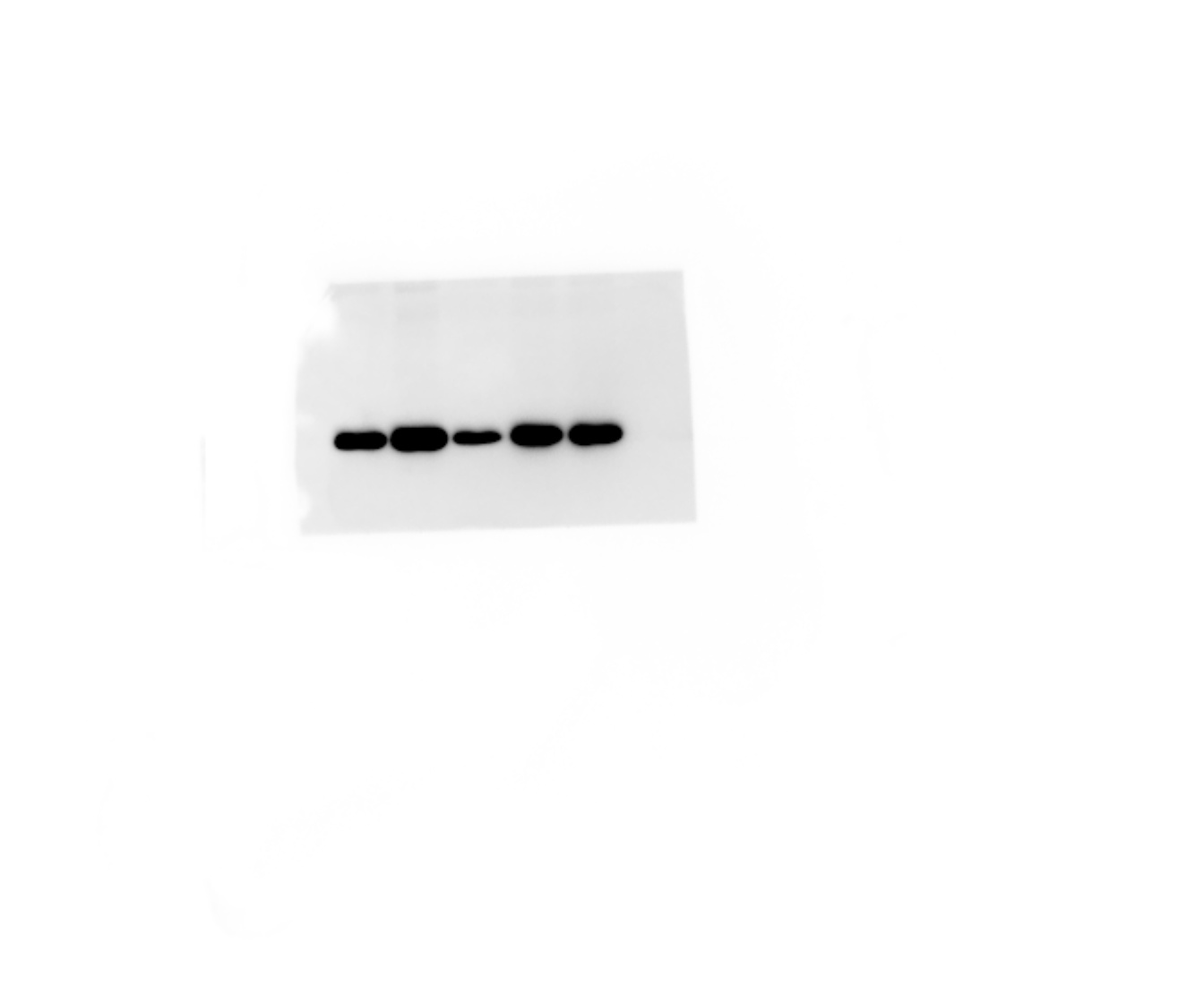


HCT8 GAPDH for Fig.7F


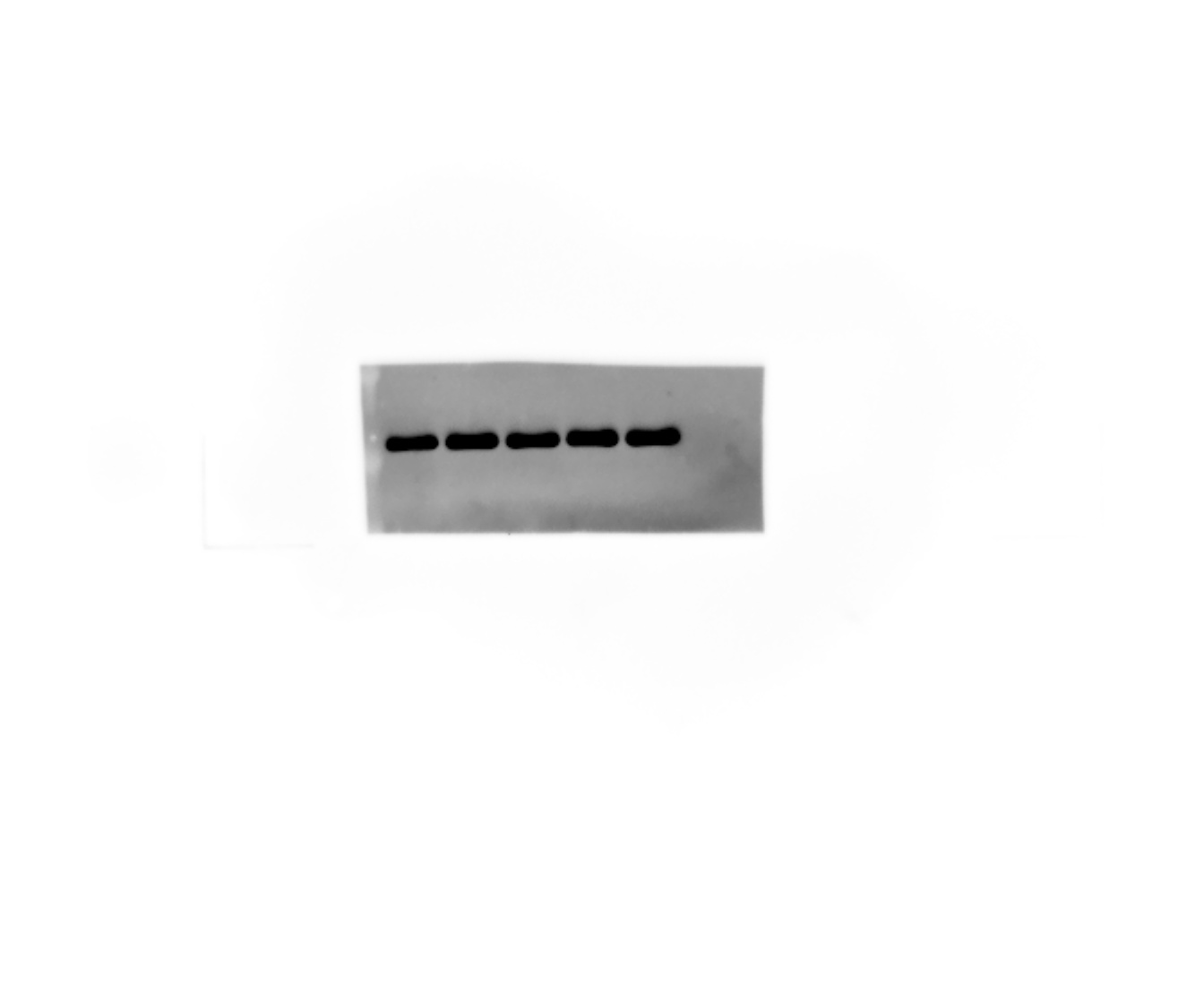

Supplement: Supplemental Material [file KBIE_A_1985259_SM7136.docx]
